# The Effects of Word Use and Personality Pathology on Depression and Suicidal Behavior in Older Adult Inpatients

**DOI:** 10.1093/geroni/igaa057.1255

**Published:** 2020-12-16

**Authors:** Hyunyoung Ellen Park, Helene Geramian, Jennifer Ho, Avner Aronov, Wing Jin Mak, Ira Yenko, Dimitry Francois, Richard Zweig, Hyunyoung Park

**Affiliations:** 1 Yeshiva University Ferkauf Graduate School of Psychology, Bronx, New York, United States; 2 Yeshiva University Ferkauf Graduate School of Psychology, The Bronx, New York, United States; 3 Yeshiva University, Ferkauf Graduate School of Psychology, Brooklyn, New York, United States; 4 Ferkauf Graduate School of Psychology, Yeshiva University, The Bronx, New York, United States; 5 Yeshiva University Ferkauf Graduate School of Psychology, Mountain View, California, United States; 6 Weill Cornell Medicine, White Plains, New York, United States

## Abstract

Few prior studies have examined whether word use is associated with personality pathology or is linked to depression and suicidal behavior in older adult inpatients. In this study, older adult depressed inpatients (N = 51; age M= 67, SD = 8.9; mean years of education = 15.49, SD = 2.6) provided narratives about high and low points in life, health challenges, and interpersonal conflicts, and completed measures of personality pathology (NEO Five Factor Inventory, Inventory of Interpersonal Problems-Personality Disorders-25), depression (Geriatric Depression Scale), and suicidal ideation or behavior (SCID-5). The Linguistic Inquiry and Word Count (LIWC) text analysis tool was used to examine the frequency of self-referential, negative emotion, or absolutist word use. After adjusting for word count, age, and occupational and marital status, the use of self-referential words in narratives about high points of life predicted suicidal behavior (OR = .563, p = .002), but word use did not predict depression or personality pathology. In regression analyses, neuroticism and maladaptive interpersonal functioning predicted depression (ß = .417, p = .002; ß = .364, p = .009, respectively) but not suicidal behavior. Together, these preliminary findings suggest that in depressed older adult inpatients, depression and suicidal behavior may have qualitatively different predictors.

